# Non-Genomic Actions of Estrogens on the DNA Repair Pathways Are Associated With Chemotherapy Resistance in Breast Cancer

**DOI:** 10.3389/fonc.2021.631007

**Published:** 2021-03-19

**Authors:** Javier E. Jiménez-Salazar, Rebeca Damian-Ferrara, Marcela Arteaga, Nikola Batina, Pablo Damián-Matsumura

**Affiliations:** ^1^ Department of Biology of Reproduction, Division of Biological Sciences and Health (DCBS), Autonomous Metropolitan University (UAM), Mexico City, Mexico; ^2^ School of Medicine, National Autonomous University of Mexico (UNAM), Mexico City, Mexico; ^3^ Monterrey Institute of Technology and Higher Education (ITESM), School of Engineering and Sciences, Monterrey, Mexico; ^4^ Nanotechnology and Molecular Engineering Laboratory, Department of Chemistry, Division of Basic Science and Engineering (DCBI), Autonomous Metropolitan University (UAM), Mexico City, Mexico

**Keywords:** chemoresistance, estrogen receptor, DNA repair mechanisms, DNA damage response (DDR), c-Src activation, breast cancer

## Abstract

Estrogens have been implicated in the etiology of breast cancer for a long time. It has been stated that long-term exposure to estrogens is associated with a higher incidence of breast cancer, since estradiol (E_2_) stimulates breast cell growth; however, its effect on DNA damage/repair is only starting to be investigated. Recent studies have documented that estrogens are able to modify the DNA damage response (DDR) and DNA repair mechanisms. On the other hand, it has been proposed that DDR machinery can be altered by estrogen signaling pathways, that can be related to cancer progression and chemoresistance. We have demonstrated that E_2_ promotes c-Src activation and breast cancer cell motility, through a non-genomic pathway. This review discusses scientific evidence supporting this non-genomic mechanism where estrogen modifies the DNA repair pathways, and its relationship to potential causes of chemoresistance.

## Introduction

The role of estrogens in the onset of breast cancer, its progression in early stages of the disease, and during invasion, has long been demonstrated (1, 2). Up to now there are few published studies on the role of these steroid hormones regarding the mechanisms of resistance to chemotherapy (3). The lack of information may be due, in part, to the fact that not all the genomic and non-genomic mechanisms of action of estrogens are yet known, as new signaling pathways continue to be reported (4–6).

Surgery and chemotherapy are the main options in the treatment of breast cancer and, when the tumors are detected in early stages, most of the cases are successful; however, when there is resistance to these treatments, the chances of adverse results increase considerably (7). Currently, resistance to chemotherapy against breast cancer is considered a multifactorial phenomenon, since it involves various mechanisms that can be activated from or through treatment (3). A wide variety of pathways have been described that induce resistance to cancer therapy, of which some stand out because of their association with estrogenic effects, in this review we will focus on three of them: A) resistance induced by altered DNA repair mechanisms [(8); B] inhibition of apoptosis through activation of PI3K/AKT (phosphatidylinositol 3-kinase/protein kinase B) pathway, which promotes cell survival (9, 10); C) the abnormal expression of proteins that regulate cell cycle progression, including p53 (tumor protein 53 KDa), MDM2 (Mouse double minute 2 homolog), and ATM (ataxia telangiectasia mutated), that are related to the regulation of cell division, and are also involved in the DNA repair mechanisms (11, 12). This article will focus on analyzing the role of estrogens and their receptors in the activation of chemoresistance mechanisms on breast cancer cells.

Estradiol (E_2_) is the most abundant and potent of the three natural estrogens, it is synthesized from cholesterol and secreted mainly by the ovary, its main function is the development and maintenance of the reproductive tract and the development of the mammary glands. For a long time, E_2_ has been implicated in the development and progression of different types of cancer (3).

The action of estrogens is mediated by two types of estrogen receptors (ER), ERα and ERβ, which belong to the superfamily of nuclear receptors that act as transcription factors. ERα contains 595 amino acids (aa), with an apparent molecular weight of 66 KDa and has three isoforms, of 53, 46, and 36 KDa (13). The canonical or classical mechanism of action begins when E_2_, due to its lipophilic nature, diffuses through the plasma membrane and interacts with an ER in the cytoplasm (either ERα, ERβ, or both), which are in monomeric forms. Once the ligand binds to the ER, its dimerization and translocation to the nucleus is favored, allowing ER-E_2_ complex bind to specific DNA sequences, known as estrogen response elements (ERE), that are present in the promoters of the target genes, inducing its transcription and translation into proteins. It is important to notice that these processes occur over a period of hours or even days, depending on the cellular environment and the type of gene to be regulated (14).

It is well known that estrogens can induce non-genomic mechanisms, which do not require the translocation of the E_2_-ER complex through the pore into the nucleus and, therefore the ER can be anchored to the cell membrane, through reversible post-translational lipid modification that involves linkage of a fatty acid chain, so they are called membrane ER alpha and beta (mERα and mERβ) (15–20). Those mER have also been described in cellular organelles, such as the mitochondria, and different tissues including liver, muscle, fat, and the β-cell of the pancreas (20, 21).

The mERα is structurally identical to the cytoplasmic ERα, since both proteins have been detected, by means of immunodetection experiments, using monoclonal antibodies directed against either the amino- or carboxyl- terminus domains, showing that they present the same molecular weight, the same electrophoretic mobility, as well as similar binding affinity to E_2_ (14, 17). Mass spectroscopy studies have shown that the difference between mERα and ERα is the presence of a molecule of fatty acid, primarily palmitate (palmitoylation) or myristate (myristoylation) that facilitates its anchorage to the plasma membrane (22, 23), the authors conclude that both, intracellular ERα and mERα are the same protein (15, 17, 24).

The non-genomic mechanisms of ER are characterized by the fact that should not enter the nucleus, and their effects are observed in short periods of time, ranging from a few seconds to minutes (16). Particularly, it has been shown that mERα can be found in the form of a monomer and that it can homodimerize, in the presence of estradiol, to activate Gα and Gβγ proteins, thus increasing the intracellular concentration of the second messengers cAMP and calcium (15). In addition, mERα homodimer can physically interact to the p85 subunit of the PI3K kinase, to the SH2 (Src Homology 2) domain of the c-Src kinase, or even activate membrane receptors with tyrosine kinase activity, such as Epidermal Growth Factor Receptor (EGFR) which stimulates the ERK (extracellular-signal-regulated kinase) or PI3K signaling pathways, increasing cell proliferation, survival, and migration of breast cancer cells (25, 26).

The non-genomic mechanism starts with the binding of E_2_ to ERα, the formation of the receptor-ligand complex induces conformational changes in ERα which leads to the dissociation of heat shock protein (HSP), and the phosphorylation of ERα in the tyrosine 537 residue (^537^Tyr), that promotes the palmitoylation of the ERα and its anchorage to the plasma membrane (25). It has been demonstrated that mERα can interact with different signaling molecules such as small G proteins or PI3K, c-Src (Rous sarcoma kinase), MAP (mitogen activated protein) kinases, to activate transcription factors downstream of the signaling cascade to regulate processes such as proliferation, differentiation, migration, cell survival, or the Epithelial-to-Mesenchymal Transition (EMT) (27).

There is evidence that mER-induced activation of c-Src can not only induce proliferation, it also favors the ubiquitination of ERα and subsequent degradation *via* proteosome (28). The loss of ERα favors EMT and tumor cell migration because ERα forms a complex with the MTA3 (Metastasis-associated protein 3) protein, in order to directly suppress the SNAIL gene. The absence of ERα or MTA3 results in aberrant expression of SNAIL and loss of E-cadherin expression, which facilitates the migration of breast cancer cells (29, 30). In addition, there is evidence that even the simple activation of the transcription factor SNAIL1 can induce transcriptional repression of ERα, demonstrating the importance of its loss in breast cancer, which correlates with poor prognosis, increased recurrence after treatment and increased incidence of metastasis (31, 32)

## Estrogens Induce Chemoresistance by Altering DNA Repair Mechanisms

**Figure 1:**
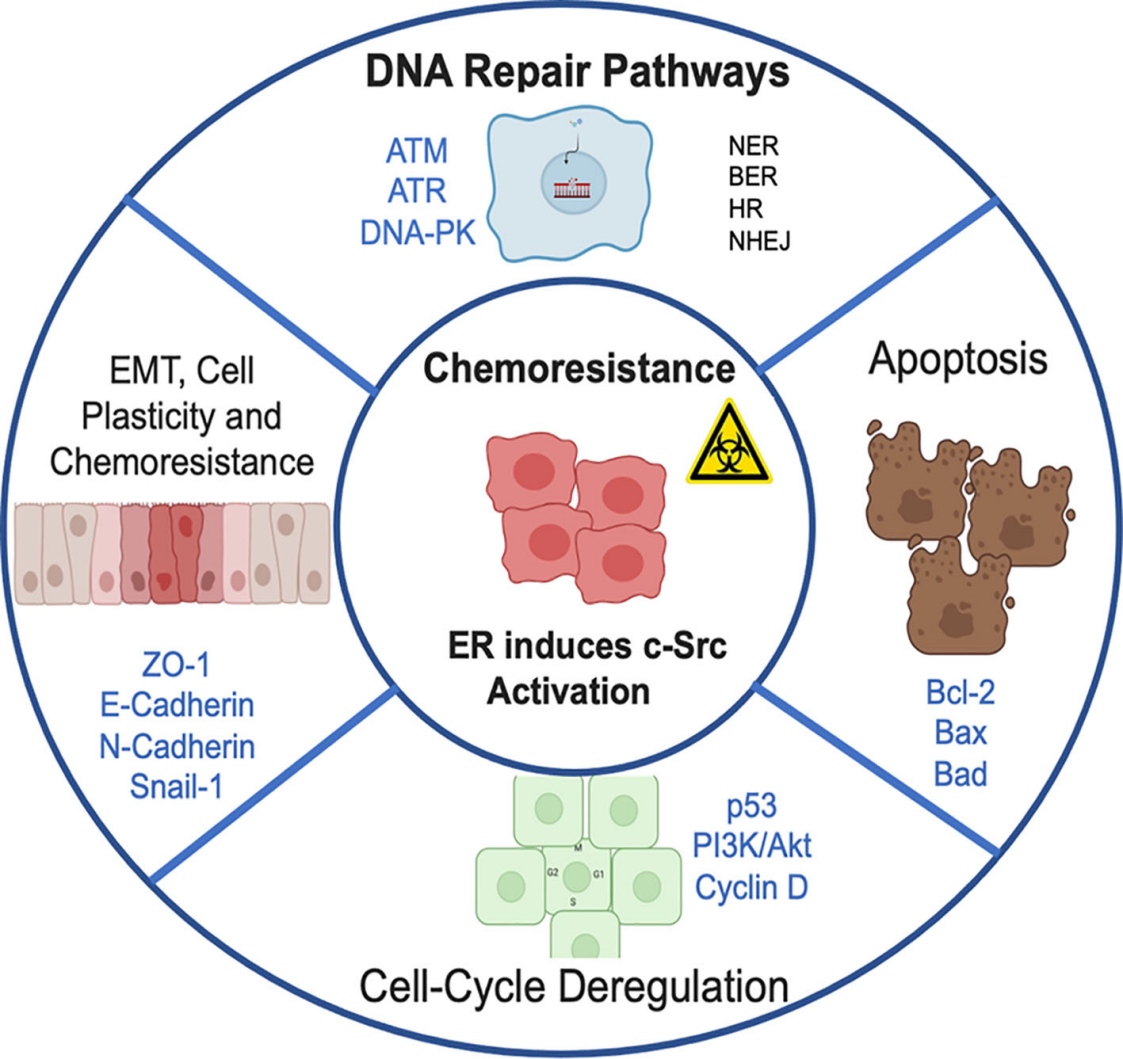
Chemoresistance pathways induced by estrogen receptor, through the direct activation of c-Src kinase. Conventional radiotherapy and chemotherapy kill most cancer cells, but there are some that can evade the cytotoxic effects of treatment and restart accelerated tumor proliferation. This promotes cancer recurrence and subsequent metastasis. Chemoresistance can be ascribed to 1) Resistance induced by altering DNA repair pathways (NER, nucleotide excision repair; BER, base excision repair; HR, homologous recombination; NHEJ, non-homologous end-joining). 2) Inhibition of apoptosis and activation of pathways involved in cell survival. 3) Abnormal expression of proteins that regulate cell cycle progression. 4) High plasticity of tumor cells with which they activate the process of EMT, epithelium-mesenchymal transition that facilitates migration and invasion to distant sites.

Various signaling pathways in cancer cells induce DNA repair and stimulate chemoresistance, avoiding the lesions generated by chemotherapy (33). DNA double-strand breaks (DSB) are highly deleterious, being sufficient to trigger cell death, or induce genomic instability by changing chromosome structure (34). Direct DSB are induced by ionizing radiation, UV light (photodynamic therapy), and free radicals (35) or certain anti-cancer drugs (36). Indirect DSB, associated with replication, occur after initial DNA damage. The most common example occurs when the replication fork meets a single-stranded break (SSB) on the leader strand of the DNA template, which can collapse, turning SSB into a DSB. Direct DSBs are repaired mainly by non-homologous end joining (NHEJ) mechanisms, while indirect DSBs are repaired predominantly by homologous recombination (HR) pathways. DNA adducts are a different form of DNA damage, which are caused by alkylating agents and are repaired by the base excision repair (BER) pathway, which removes a base or short sequence of nucleotides where the damaged base is found. The nucleotide excision repair (NER) pathway removes a single strand of damaged DNA with a length of 24–30 base pairs. Another important DNA repair pathway is the direct repair (DR) pathway, which can repair damaged DNA without removing the damaged base (37). Recent studies have documented interactions between DNA damage repair pathways and genomic and non-genomic mechanisms of ER (38, 39). Diverse studies in patients with ER-positive breast cancer, showed that the DNA repair capacity was reduced by 50% compared to free-disease women, effect that was associated to the decrease in the NER pathway (40–42) (**Figure 1**).

DNA damage response activates diverse repair mechanisms by inducing signals through three major effector kinases, ATM, ATR (ataxia telangiectasia and Rad3-related), and DNA-PK (DNA-dependent protein kinase). On one hand, ATM and DNA-PK recognize DSB, while ATR responds to SSB in the replication forks. The downstream signaling of ATM, ATR, and DNA-PK, involves a large amount of proteins; however, there are key effector proteins including CHK1 (Checkpoint kinase 1), BRCA1 (Breast Cancer gene 1), p53, and MDM2 that signal other targets, such as BRCA, cyclin D, pRb (retinoblastoma protein), and p21 (21 kDa protein), in addition to the control points of the cell cycle and the machinery that regulates the apoptosis process (43, 44).

There are different molecular mechanisms through which estrogens and ERα contribute to the radioresistance and chemoresistance. Several reports have shown that ERα could regulate the expression of ATM, a phosphotransferase of serine residues whose main function is to activate different signals in response to DNA damage. The proposal indicates that ERα does not exert directly to the promoter of the ATM gene, but ERα targets the miRNAs (miR-18a and 106a) in order to inhibit ATM expression. It has also been demonstrated that the expression of miR-18a and 106a is significantly reduced in tissues of patients diagnosed with ERα-positive breast cancer (45).

**Figure 2:**
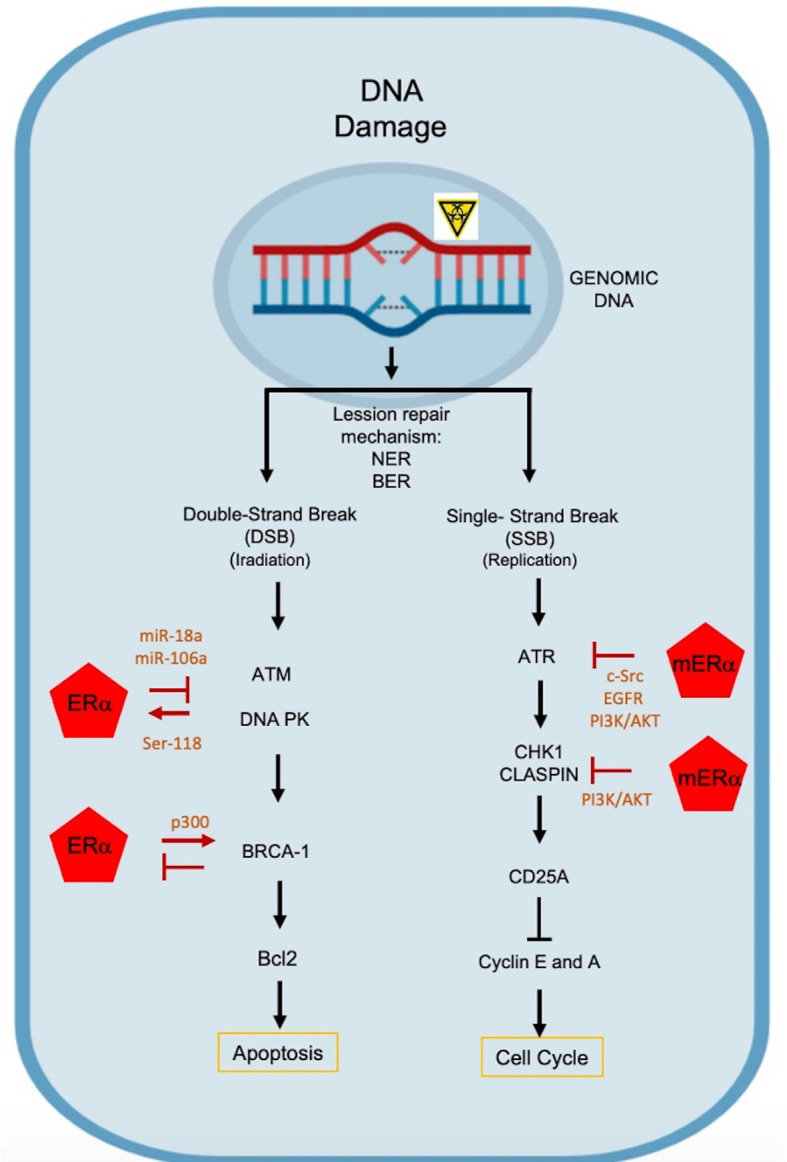
Molecular mechanisms by which estrogens mediate chemoresistance. DNA alterations induce responses to DNA damage (DDR), activating different DNA repair mechanisms: NER, nucleotide excision repair; BER, base excision repair. DNA double-stranded breaks activate ATM and PK DNA effector kinases. ATM is repressed by ER through miR-18a and miR106a, and PK DNA phosphorylates ERα in the 118Ser residue, improving its transcriptional activation and preventing its degradation *via* proteasome. BRCA-1 represses ERα activity and ER regulates BRCA1 transcriptionally. Single-stranded DNA breaks activate the ATR kinase, which is blocked by non-genomic mREα signaling that activates the PI3K/AKT pathway through c-Src kinase and EGFR. On the other hand, CHK1 and CLASPIN are repressed, inducing the activation of the PI3K/AKT pathway.

Like other steroid hormone receptors, ERα is a substrate of DNA-PK, a key component of the NHEJ, that phosphorylates serine and threonine residues. Estrogens promote the formation of the DNA-PK/ER complex, the phosphorylation of ^118^Ser in ERα, that is required for its transcriptional activation and maintains its stability. This report has been corroborated by the fact that inhibition of DNA-PK promotes ERα degradation *via* proteasome (46, 47) (**Figure 2**).

ATR protein, a Serine/Threonine (Ser/Thr) kinase, is activated by alterations in the replication process, and requires its binding to ATRIP (ATR Interacting Protein) and TopBP1 (DNA topoisomerase II binding protein 1) proteins. The latter is a phosphoprotein that is specially regulated by Akt. Several studies indicate that E_2_ induces phosphorylation of TopBP1 at the site of interaction with AKT. When TopBP1 is phosphorylated in ^1159^Ser, its association to ATR is avoided and their functions are avoided (48). It has been proposed that E_2_ can block the activation of ATR, after DNA damage, through a mechanism mediated by the ERα-Src, as it can activate the PI3K/AKT pathway (**Figure 2**). It is also probable that the mERα-Src complex induce the activation of EGFR and PI3K/AKT pathway to restrict ATR signaling in the tumor process (48, 49).

Another proposed mechanism is that mER-E_2_ complex inhibits the activation of Chk1, a Ser/Thr kinase that is required for cell cycle arrest, since it is activated at checkpoints in response to DNA damage. Alternatively, Claspin (Chk1-interacting protein) binds directly to the kinase domain of Chk1, inducing cell cycle arrest (50). Therefore, E_2_ can induce the ER/c-Src or ER/c-Src/EGFR pathways in order to activate the PI3K/AKT pathway, where AKT inhibits the phosphorylation of the ^345^Ser in Chk1 and precludes the formation of the Chk1/Claspin complex, preventing the cell cycle arrest (43).

BRCA1 is a human tumor suppressor protein related to estrogens actions, responsible for inducing DNA repair mechanisms and it is an effector downstream of the signaling cascade, recruited at sites of DNA damage. It works directly on homologous repair mechanisms, influences cell cycle arrest and other repair pathways. There is evidence that BRCA1 modifies estrogen-mediated tumor progression, as increased E_2_-ER signal accelerate preneoplasia and cancer development in the absences of BRCA1, favoring breast de-differentiation and tumorigenesis (51, 52). Some reports suggest that BRCA1 represses the transcriptional activity of ERα, the mechanism suggests the physical interaction is between the amino-terminal of BRCA1 and the AF-1 domain of ERα, the binding has not been fully demonstrated; however, the carboxyl-terminal (where the repression domain is present) is required to achieve the repression of ERα activity, since mutant BRCA1 proteins that lack this domain do not inhibit Erα (53, 54). While BRCA1 suppresses the function of ERα, estrogens promote BRCA1 transcription (55–57).

## Estrogens Induce Resistance Through Improper Expression and Activation of the Proteins That Control the Cell Cycle

Radiation therapy and chemotherapy result in delayed progression of cell cycle stages. This occurs through activation of the DDR, that activates different control points (Chks) at different stages of the cell cycle. These checkpoints are found in G1/S, S, early G2, and late G2 phases (11, 58). In tumor cells, one or more of these checkpoints are disabled due to genetic changes and other alterations that occur during tumorigenesis. Cells in G2/mitosis phase are more sensitive to radiation and those that are in late S-phase are more radiologically resilient (59). It has been demonstrated that Chk 1 kinase activation is essential for stopping the cell cycle in G1/S or G2/M checkpoints, in response to DNA damage (60). The G1/S checkpoint pathway is primarily regulated by two key effectors, the transcription factor p53 and the cell division cycle phosphatase 25A (Cdc25A). p53 is a tumor suppressor and DNA damage sensor that can activate different cellular processes, such as cell cycle arrest, DNA repair, and even apoptosis. The main function of Cdc25A is the inhibition of the CDK2 (cyclin dependent kinase)-cyclin A/E and CDK1-cyclin B complexes (61). On the other hand, p53 has a bidirectional relationship with the ER that affects both, its expression and function. The TP53 gene is transcriptionally repressed by ERα, due to the recruitment of the nuclear coregulators NCoR (nuclear coregulators), including SMRT (silencing mediator for retinoid and thyroid hormone receptor) and histone deacetylases HDAC (histone deacetylases) (62). It is known that ERα interacts physically with p53 inducing functional repression of the p53 transcriptional activity. In addition, estrogens induce the formation of the ERα/p53 complex, associate directly at the p21 promoter to block its transcription, avoiding the arrest of the cell cycle and promoting radioresistance (63). It has also been demonstrated that the ERα/p53 complex can bind to the MDM2 protein, a ubiquitin E3 ligase, which ubiquitinates the carboxyl-terminal domain of p53, inhibiting its function. The ERα/p53/MDM2 complex negatively regulates the activity of ERα through ubiquitination, induced by the ubiquitin ligase of MDM2 (64).

**Figure 3:**
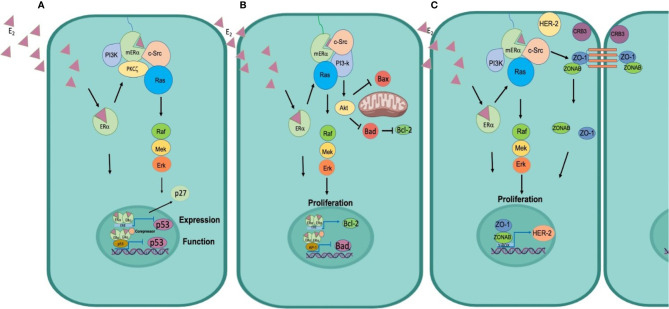
Mechanisms of estrogen-induced resistance and signaling of its receptors. **(A)** Control of the cell cycle. ER functions as a transcription factor to regulate p53 expression, in addition ER can physically interact with p53 to inhibit p53 function. Moreover, it signals through the c-Src kinase and switches on the PI3K and Ras/MAPK pathways to promote activation of Cyclin D and release the p27 repressor from the nucleus to promote cell cycle progression. **(B)** Inhibition of apoptosis ER functions as a transcription factor to induce the expression of the anti-apoptotic protein Bcl2 and functions as a transcriptional co-repressor of Bad to inhibit its synthesis. Furthermore, mERα associates with c-Src, to activate the PI3k/Akt pathway, Akt phosphorylates Bad to preclude the formation of Bad/Bad homodimer and block the release of cytochrome C and thus the process of apoptosis. **(C)** Cellular plasticity promotes TEM. E2 diffuses through the plasma membrane binds to mER, induces phosphorylation of c-Src, its associated with ZO1, and forms the p-Src/ZO-1 complex, that favors the dissociation of the Tight Junctions and promotes that ZO1 and ZONAB are translocated to the nucleus, inducing the transcription of HER-2, which triggers EMT and migration of tumor cells.

Non-genomic mechanisms of estrogens have been described in the regulation of the cell cycle. This mechanism begins when E_2_, due to its lipophilic properties, diffuses through the plasma membrane, and binds to the mERα (44). The activation of the receptor facilitates the interaction of mERα with the SH2 domain of c-Src kinase, and with the p85 alpha subunit of PI3K, the formation of the mER-Src/PI3K complex triggers the activation of mERα *via* Akt and PKCζ (Protein kinase C, zeta). Activation of Akt increases the transcription of cyclin D1, and PKCζ controls the association of Ras GTPase to the mERα/Src/PI3-K complex, inducing the Raf/MEK/ERK pathway, favoring the translocation of ERK to the nucleus and the consequent release of p27 outside the nucleus, stimulating the G1 to S transition of the cancer cells (65) (**Figure 3A**).

A different non-genomic mechanism reported for E_2_, in cells where the presence of ER or mER is undetectable, is through the G Protein-coupled Estrogen Receptor (GPER) that belongs to the superfamily of seven transmembrane domains receptors. GPER plays a critical role in the signaling of steroids in triple-negative tumors. GPER mechanism starts it binds to E_2_, inducing conformational changes that activates the trimeric G-protein that is coupled to the receptor, which induces the activation of c-Src kinase and the formation of the c-Src/PI3-K complex that favors the activation of Akt and PKCζ pathways, where Akt increases the transcription of cyclin D1, and PKCζ actives the Ras/Raf/MEK/ERK pathway, promoting the G1/S transition in cancer cells (66).

## Estrogens Induce Resistance by Inhibiting Apoptosis

Programmed cell death, a process known as apoptosis, is highly regulated as it plays a critical role in development and homeostasis by eliminating unnecessary cells. The process of apoptosis is carried out by the activation of two major signaling pathways: the intrinsic pathway, which proceeds in the mitochondrion, sensing irreparable DNA damage, and inducing the formation of a homodimer of the members of the pro-apoptotic protein superfamily (Bax, Bak, Bad, Bid, Puma, Blim). Homodimers of pro-apoptotic proteins induces the presences of protein-permeable pores in the outer mitochondrial membrane that release cytochrome c, and the activation of the initiating caspases (caspase 9) and the effector caspases (caspase 3). The extrinsic pathway occurs at the cellular membrane level, its signaling starts with the activation of the death receptors Fas and the ligand inducer of TRAIL (TNF [tumor necrosis factor]-related apoptosis protein); the ligand-receptor interaction induces the activation of caspase 8 and trigger effector caspases 3, 6, and 7, being caspase 3 the most frequently activated (67). The onset of apoptosis is the last mechanism that protects cells from irreparable DNA damage. To prevent the uncontrolled proliferation of cells, p53 induces the expression of proapoptotic genes such as FAS-R, BAX, PUMA, and NOXA (68, 69).

One of the most important functions of DDR is to stop proliferation by activating cell cycle control points before inducing death by apoptosis. In the cell cycle arrest, E_2_ play a relevant role in resistance induction since some reports indicate that the ERα stimulates cell cycle progression through positive transcriptional regulation of cyclin D, since ERα interacts to an AMPc-response element (CRE) present in the cyclin D gene promoter, and induces the synthesis of the *c-myc* oncogene which controls the expression of cyclin D1 (70). The induction of c-Myc by E_2_ is also generated by the binding of ERα to an ERE, present in the promoter region of the *c-myc* gene (60, 71). The E_2_-ER rapidly activates the cyclin E/CDK2 complex accelerating the transition from G1 to S; furthermore, ER negatively regulates the expression of the p21 inhibitor to induce the progression of the cell cycle (72). At the same time that the cell cycle is stopped, E_2_ must also block the apoptosis process, inducing the expression of some anti-apoptotic proteins such as Bcl-2 and BclXL (73, 74). It has been reported that ER can positively regulate the transcription of Bcl2 and BclXL by direct binding to ERE sites present in their promoters or, as in the case of Mcl1, through Sp1 sites. ER can additionally function as a transcriptional repressor of proapoptotic genes such as Bad, Bak, Bid, or Puma to inhibit the intrinsic pathway of apoptosis (75–77).

In addition to the genomic mechanisms, estrogens can induce effects through non-genomic mechanisms (mERα) and are independent of ER-mediated transcription. As mentioned before, ERα can interact with several proteins in this process, including c-Src, the p85 alpha subunit of PI3K, caveolin 1, EGFR (epidermal growth factor receptor), IGFR1 (insulin-like growth factor receptor 1 IGFR1), and HER2 (human epidermal growth factor receptor 2) (78). The formation of this complex rapidly increases the activity of PLCζ, and signal crossover by activating the MAPK and the PI3K/Akt pathways. Akt mediates the phosphorylation of Bad pro-apoptotic protein, Bad phosphorylation inhibits dimers formation in the mitochondria and thus prevents the release of cytochrome c, therefore favoring cell proliferation and survival (14, 47) (**Figure 3B**).

## Estrogens Induce Resistance by the Plasticity of the Tumor Cells That Favor EMT

Plasticity of tumor cells is associated with the ability they acquire to undergo phenotypic transitions in response to exposure to different stimuli or to specific microenvironmental factors. Dedifferentiation is a process by which tumor cells change their phenotype, giving them plasticity that facilitates the EMT, which is a key process in cancer progression because it is activated in the early stages. This process is before to metastasis and, in order to understand EMT details, it has been divided into three main phases: 1) change in gene expression (transformation to neoplastic cells), 2) loss of epithelial phenotype, 3) gain of mesenchymal phenotype. A key process is the decrease in cell-cell adhesion strength due to the disassembly of the proteins that form the intercellular junctions, the expression of these proteins is lost, changing its intracellular location, particularly E-cadherin, occludin, and cytokeratins. The latter is associated to the reorganization of the actin cytoskeleton that causes the morphological change of the epithelial cells (79). The lack of the limits established by the tight junctions around the cells allows that proteins at the baso-lateral region migrate to the apical zone, which causes the loss of the cellular polarity. On the other hand, the paracellular permeability increases and the transepithelial resistance decreases, which denotes the loss of the function of the intercellular junctions. Finally, proteins of the mesenchymal phenotype are synthesized, such as N-cadherin, vimentin, the transcription factor Snail and the fibroblast specific protein (FSP1), that contributes to the fibroblast-shape, lower expression of proteins associated to the epithelial phenotype, loss of morphogenesis, and increase in the expression of mesenchymal phenotype markers (80–82), that are associated to cell adhesion and invasion.

These changes contribute to the acquisition of a frontal polarity (front to rear) that generates a migration front, characteristic of mesenchymal cells (83). Due to the heterogeneity of the cell populations that occur in cancer, it should be noted that not all stages of EMT can be carried out on all cancer cells, nor are all the markers expressed, making it more difficult to detect them.

Development of resistance to cancer treatments remains as a major impediment in medical oncology. Resistance may not only precede but may also arise because of therapy. The use of doxorubicin, a cytotoxic drug commonly used in clinical practice, may promote resistance in HCT colon cancer cells through the activation of TGFβ signaling and phosphorylation of Smad2 and Smad3. This enhances the expression of Snail, Slug, vimentin, and N-cadherin, mesenchymal phenotype marker proteins, and decrease epithelial markers such as E-cadherin and occludin (84). One of the main inducers of these changes are estrogens through c-Src signaling. Our research has demonstrated the role of E_2_ in EMT by triggering c-Src, the formation of the p-Src/ZO-1 (Zonula occludens protein) complex that promotes the dissociation of ZO-1 and ZONAB (ZO-1-associated nucleic acid-binding protein) proteins from tight junctions, and higher expression of mRNA encoding for HER2. These changes were correlated with decreased expression of the epithelial markers occludin and protein crumbs homolog 3 (CRB3) and increased synthesis of N-cadherin and the transcription factor SNAIL, that induced E_2_-dependent migration of MCF-7 and T47D breast cancer cells. Incubation with the ER antagonist Fulvestrant precluded the effects of E_2_ on c-Src phosphorylation, p-Src/ZO-1 complex formation, ZO1/ZONAB nuclear translocation and MCF-7 cell migration. Our findings suggested that E_2_ promotes tight junction dissociation during tumor progression and increases MCF-7 cell migration (27) (**Figure 3C**).

There is *in vivo* and *in vitro* evidence that supports the fact that estrogens contribute to tumor chemoresistance, through the activation of c-Src. It has been shown that c-Src activation can be considered a marker of tumor progression. Ke and collaborators identified high concentrations of phosphorylated c-Src in tyrosine 419 (^419^Y-cSrc) in serum samples from patients with nasopharyngeal carcinomas (NPC), which were associated with an unfavorable prognosis in parameters such as survival, disease-free period, and survival free of distant metastases. In the same study, blocking or inactivating c-Src in PCN cell lines decreased cell viability, colony formation, cell migration, *in vitro* invasion, and *in vivo* metastasis (85). Studies on 50 primary ERα+ and 200 ERα- breast cancer tumor samples demonstrated that E_2_, induces activation of c-Src kinase and rapidly stimulates the ubiquitination of ERα and its subsequent degradation, *via* proteosome. In the same report, but in cell lines, blocking proteosome activity was shown to increase ERα levels (28).

Finally, another study shows that deregulation in the activation of c-Src kinase in solid tumors favors the epithelium-mesenchyme transition and chemoresistance in breast cancer cells, favoring their migration and invasion (86).

## Conclusions

Several studies have examined the role of estrogens, and their receptors, in chemoresistance; however, their molecular mechanisms of action remain elusive. Its association with DNA damage and repair mechanisms are just beginning to be studied, especially those related to kinase activation such as c-Src, EGFR, HER2, ERK, or PI3K/AKT.

One mechanism by which estrogens favor the proliferation of breast cancer is through the induction of DNA damage, although they can also contribute to chemoresistance by altering the mechanisms of DNA repair, preventing apoptosis, or deregulating the control mechanisms of the cell cycle, most of them induced by the activation of c-Src.

Considering that ERα is expressed in more than 60% of the breast tumors, estrogen-mediated chemoresistance has become a challenge to find better treatments. For this reason, knowing and understanding the pathways through which estrogens activate drug resistance will allow the design of better therapeutic strategies.

Overall, the above-mentioned research suggests that estrogens and their genomic and non-genomic signaling contribute to tumor chemoresistance and a better understanding of the molecular mechanisms of DNA repair, cell cycle regulation, apoptosis inhibition, and cell plasticity that induces EMT could improve the development of novel combination therapies or tumor markers.

## Author Contributions

JJ-S and PD-M conceived of the presented idea. PD-M wrote the manuscript with support from JJ-S, NB, MA, and RD-F. All authors contributed to the article and approved the submitted version.

## Funding

This work was supported by CONACYT, through a postdoc grant to JJ-S (Doctorate in Biomedical Sciences, School of Medicine, UNAM, Mexico), CBS-UAM, Mexico to PD-M, and CONACyT-Mexico to NB (grant number INFR-2016-01-270227).

## Conflict of Interest

The authors declare that the research was conducted in the absence of any commercial or financial relationships that could be construed as a potential conflict of interest.
